# The role of cerebral-cognitive reserve in the birth of a child with Alzheimer’s in late-life individuals

**DOI:** 10.1192/j.eurpsy.2024.1301

**Published:** 2024-08-27

**Authors:** A. Sidenkova

**Affiliations:** Psychiatry, Ural State Medical University, Yekaterinburg, Russian Federation

## Abstract

**Introduction:**

The modern understanding of AD allows us to consider it through the constructs of “vulnerability” and “stability” of the brain in relation to the pathological effects of neurodegeneration. To describe the resistance of the brain to a developing lesion due to a pathological process, the concept of “reserve” is proposed.

**Objectives:**

A systematic review of scientific studies was conducted.

**Methods:**

The review includes an analysis of full-text literature sources.

**Results:**

Resilience models based on reserves are described, which can be broadly divided into cerebral and cognitive reserve models. The quality of the brain substrate underlies the cerebral reserve. Its role and power are determined by the ratio of healthy/affected neurons, the integrity of synaptic connections, and the size of the brain/ It seems to us that the conditions that promote or hinder the functioning of the brain should also be taken into account when characterizing the cerebral reserve. Cognitive reserve is determined by the phenomena of mental processes and functions. It includes the individual’s involvement of the individual in various cognitively stimulating activities throughout life. Cognitive reserve plays a decisive role when it comes to determining the effectiveness of the activation of additional areas or the implementation compensatory strategies, behaving more flexibly and dynamically than the passive threshold. Brain and cognitive reserve models cannot be considered mutually exclusive. They reflect different categorical levels: substrate and functional. The cerebral reserve system is the morphological basis of the cognitive reserve. In fact, we can talk about a single cerebral-cognitive reserve.

**Conclusions:**

The reserve concept states that there are individual differences in the adaptability of the functional processes of the brain that allow some people to cope with age-related and disease-related brain changes better than others. The reserve plays a protective role, postponing clinical manifestations and ensuring that adequate cognitive functioning is maintained. There is a transition from the protective role of the reserve to the compensatory function. Even after anatomical signs of brain damage are observed, the time to clinical conversion can be modulated depending on the volume of the reserve. The protection mechanisms underlying the reserve concept are partially controllable, which allows building strategies for correcting cellular homeostasis, brain functions, behavioral and cognitive patterns. Understanding the mechanisms of aging and the determinants of life expectancy will help reduce age-related morbidity and promote healthy aging.

**Disclosure of Interest:**

None Declared

